# Nanoparticle-Driven Modulation of Mucosal Immunity and Interplay with the Microbiome

**DOI:** 10.4014/jmb.2504.04033

**Published:** 2025-06-12

**Authors:** Won Jung, Young Min Son

**Affiliations:** Department of Systems Biotechnology, Chung-Ang University, Anseong-si 17546, Republic of Korea

**Keywords:** Mucosal immunity, microbiome modulation, nanoparticle-based delivery, prebiotic nanoparticle, probiotic nanoparticle, synbiotic nanoparticle

## Abstract

Mucosal surfaces are dynamic immunological interfaces that play a critical role in maintaining host defense and microbial homeostasis. Disruptions in the interaction between the mucosal immune system and its commensal microbiota have been associated with the onset of several diseases, including inflammatory bowel disease, asthma, and bacterial vaginosis. This review examines recent advances in nanoparticle (NP)-based strategies aimed at modulating mucosal immunity and restructuring microbial communities. It highlights how organic and inorganic NPs such as polysaccharide-based carriers, lipid NPs, and metallic nanomaterials enhance the delivery and stability of probiotics, prebiotics, and synbiotics, and facilitate targeted immunomodulation across gastrointestinal, respiratory, and female reproductive mucosal tissues. NP-based strategies are particularly emphasized for their ability to penetrate mucus barriers, facilitate microbial colonization, modulate cytokine activity, and enhance the restoration of epithelial barrier function. Disease-specific applications, including NP-based therapies for colitis, respiratory inflammation, and vaginal dysbiosis, are also discussed. In addition, this review outlines current challenges related to biosafety, targeting specificity, and clinical translation, and suggests future directions for research. Altogether, NP platforms offer a promising avenue for the precise modulation of mucosal immunity and microbiota, with significant potential in the prevention and treatment of mucosal-associated diseases.

## Introduction

Mucosal surfaces are fundamental interfaces between the host and its external environment, forming the first line of defense while facilitating essential physiological functions. Located in the gastrointestinal (GI), respiratory, and female reproductive tracts, these surfaces not only act as barriers against pathogens but also support symbiotic relationships with commensal microorganisms [1, 2]. The mucosal areas are equipped with complex networks of physical, chemical, and immune mechanisms that defend against pathogenic microbial threats while facilitating essential physiological functions [3].

The mucosal immune system is uniquely positioned to balance protective immunity with immune tolerance [4]. It relies on an intricate interplay of epithelial barriers, innate immune defenses, and adaptive immune responses to recognize and eliminate pathogens while maintaining tolerance to commensal microorganisms and dietary antigens [5]. Disruption of this delicate balance may result in immune dysfunction, contributing to the onset of inflammatory disorders, allergic reactions, and susceptibility to infections [6].

The mucosal microbiome encompasses groups of microbes and their genetic material forming a complex and dynamic ecosystem of microorganisms inhabiting mucosal surfaces that closely interact with the immune system [7]. Predominantly composed of bacteria, the microbial community plays a critical role in modulating immune responses, preserving epithelial barrier function, and regulating host metabolic pathways [8]. Interestingly, certain microbial taxa within the general microbiota have been shown to influence the regulation of mucosal immune systems including adaptive, innate, and cell-autonomous immune responses [7]. However, disruption of the balance of the microbiome composition known as dysbiosis, characterized by reduced microbial diversity or the over proliferation of pathogenic species, is increasingly implicated in the pathogenesis of mucosal and systemic disorders, including inflammatory bowel disease (IBD), asthma, and bacterial vaginosis (BV) [9, 10].

Recent advances in nanotechnology have provided novel tools to address the challenges associated with mucosal immunity and microbiome modulation [11]. Nanoparticles (NPs), characterized by their nanoscale dimensions and highly tunable physicochemical properties, demonstrate significant potential in targeting the mucosal microenvironment [12]. Owing to their unique physicochemical properties, NPs represent promising platforms for the design of drug delivery systems and the development of therapeutic strategies aimed at restoring microbial homeostasis [13]. Nevertheless, direct evidence for the role of nanoparticle-based strategies in mucosal microbiome regulation remains largely unexplored.

This review explores the distinct roles of mucosal immunity, the microbiome, and NPs, examining their individual contributions and interconnections. We suggest that NPs, by targeting microbial imbalances and modulating immune responses, offer promising avenues for supporting microbial homeostasis and mitigating the effects of dysbiosis-associated conditions.

## Overview of Mucosal Immunity

Mucosal surfaces represent the first site of interaction between the host and its external surroundings [14]. The mucosal surfaces fulfill a dual role by both defending against pathogen invasion and fostering symbiotic interactions with commensal microorganisms that reside on mucosal sites [15]. The mucosal immune system relies on a coordinated network of physical, chemical, and immunological defenses to inhibit pathogen entry while ensuring the preservation of critical processes such as nutrient absorption, gas exchange and maintenance of reproductive tract integrity [16]. The major components of the mucosal immune system consist of diverse immune cell populations, epithelial cells, mucus layers, and a range of antimicrobial mediators [17]. Together, these elements form a dynamic barrier that ensures a balance between immune protection and tolerance to non-pathogenic antigens, maintaining microbial and tissue equilibrium [18].

### Mucosal Epithelial Cells

Epithelial cells form a structurally continuous barrier maintained by tight junctions, which regulate selective permeability to support critical physiological functions while restricting pathogen entry [19]. In addition to their barrier function, epithelial cells actively interact with microbiota, which play a critical role in maintaining barrier integrity and modulating immune responses [20]. For instance, epithelial cells respond to microbial metabolites, such as SCFAs (short-chain fatty acids) including propionate, acetate, and butyrate, which are produced by microbiota, enhance tight junction integrity and promote anti-inflammatory pathways [21]. Beyond their structural role, epithelial cells produce antimicrobial peptides (AMPs) including defensins, cathelicidins, and lactoferrin, which exhibit potent antimicrobial activity against a broad spectrum of pathogens [22]. For instance, epithelial cells recognize bacteria through Toll-like receptors (TLRs) or NOD-like receptors, triggering signaling pathways that produce AMPs and cytokines while avoiding excessive inflammation to maintain homeostasis [23].

In contrast, pathogenic bacteria exploit epithelial cells to facilitate infection. Pathogens such *as* *Helicobacter pylori*, *Escherichia coli*, and *Pseudomonas aeruginosa* disrupt tight junctions by secreting virulence factors, weakening the epithelial barrier and allowing microbial translocation [24]. For instance, enterohemorrhagic *E. coli* produces Shiga toxin, which damages epithelial cells and disrupts the intestinal barrier [25]. Additionally, pathogens such as *E. coli*, *Salmonella* and *Shigella*, induce epithelial cell apoptosis or hijack endocytic pathways to gain intracellular access, further compromising barrier integrity [26].

### Innate Immunity

Innate immune cells including dendritic cells (DCs), macrophages, innate lymphoid cells, and natural killer (NK) cells, are strategically distributed within mucosal tissues, each performing distinct and complementary roles in host defense [27].

Macrophages are abundant in mucosal tissues, playing key roles in pathogen clearance, apoptotic cell removal, and tissue repair [28]. They balance pro-inflammatory and anti-inflammatory responses to maintain immune homeostasis [29]. Within the GI tract, intestinal macrophages of the M2 phenotype secrete anti-inflammatory cytokines, such as IL (interleukin)-10, IL-4, and transforming growth factor beta (TGF-β) to mitigate excessive inflammatory responses and facilitate immune tolerance to commensal microbiota, thereby preserving the integrity of the gut barrier [30]. In the respiratory system, alveolar macrophages clear pathogens and particulates while producing IL-10 to prevent inflammation-induced damage to the lungs. Alveolar macrophages also coordinate tissue repair after injury or infection [31].

DCs, as the major antigen-presenting cells (APCs), capture mucosal antigens and migrate to lymph nodes to activate T cells and initiate adaptive immunity [32]. In the intestinal mucosa, they induce immune tolerance to dietary antigens and commensal microbes, preventing aberrant immune activation [33]. In the female reproductive mucosa, microbes such as *Lactobacillus* promote DC-induced regulatory T cell (T_reg_) differentiation, suppressing excessive inflammation and maintaining immune homeostasis [34].

### Adaptive Immunity

The adaptive immune system in mucosal tissues is highly specialized to provide long-term, pathogen-specific immunity while maintaining tolerance to non-pathogenic antigens [35]. This coordinated interaction shapes antigen-specific immune responses. This dual functionality enables the immune system to effectively combat infections while preserving tissue integrity by minimizing excessive or inappropriate inflammatory responses [36].

T cells in mucosal immunity encompass diverse subtypes, each specialized to address distinct immunological challenges [37]. These include CD4^+^ helper T cells, CD8^+^ cytotoxic T cells (CTLs), γδ T cells, and others [38]. Collectively, these subtypes coordinate a highly adaptive immune response tailored to the specific needs of mucosal tissues.

T helper cells, known as CD4^+^ T cells, comprise several subtypes, including T helper 1 (T_H_1), T helper 2 (T_H_2), T helper 17 (T_H_17), and T_reg_ cells. These subsets contribute respectively to intracellular pathogen clearance, antiparasitic immunity, induction of antimicrobial peptides, and regulation of excessive immune responses [39].

CTLs are fundamental in targeting and eliminating infected or abnormal epithelial cells in mucosal tissue by recognizing pathogen-derived peptides on major histocompatibility complex (MHC) class I from DCs and releasing perforin and granzyme [40].

In conclusion, the mucosal immune system orchestrates a complex interplay of physical barriers, innate defenses, and adaptive responses to protect against pathogens while maintaining tolerance to commensals and non-threatening antigens [41]. This balance is critical for preserving tissue integrity across the GI, respiratory, and reproductive tracts [42], highlighting its central role in health and its potential as a target for advanced therapeutic strategies.

## Mucosal Microbiome

**Fig. 1:**
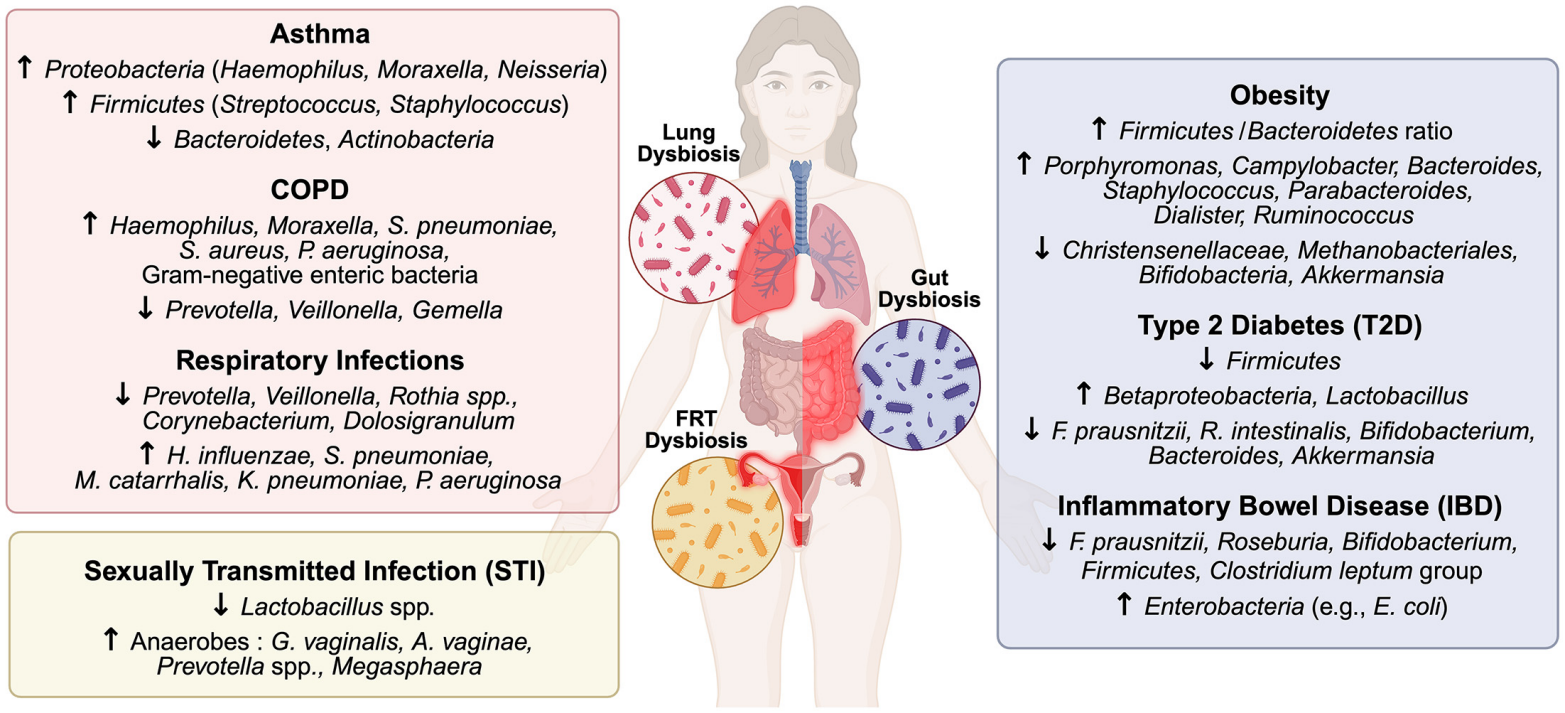
Representative patterns of mucosal dysbiosis across disease conditions. Site-specific microbial shifts observed in the gut, respiratory tract, and female reproductive tract under various disease conditions. Dysbiosis is represented by the relative enrichment or depletion of microbial taxa associated with obesity, type 2 diabetes, inflammatory bowel disease, asthma, chronic obstructive pulmonary disease, respiratory infections, and sexually transmitted infections.

Technological progress in metagenomics and 16S rRNA-based sequencing has greatly enhanced insights into the mucosal microbiome [43]. These methodologies have enabled the comprehensive identification and characterization of non-culturable microorganisms that were previously undetectable using conventional culture-based approaches [44]. Bacteria, the dominant members of the mucosal microbiome, coexist with diverse microorganisms such as archaea, fungi, viruses, and protozoa, residing on mucosal surfaces [45]. These microorganisms interact with microbial metabolites and the surrounding microenvironment to support critical host functions and microbial homeostasis [46]. The mucosal microbiome regulates key host physiological processes including nutrient metabolism, immune modulation, and epithelial barrier defense [4], thereby maintaining microbial equilibrium. Disruptions in this balance, referred to as dysbiosis, can lead to immune dysfunction and increase the risk of various mucosal and systemic diseases [47] (Fig. 1).

### The Gut Microbiome

The gut microbiome, hosting over a trillion microorganisms, is the most extensively studied mucosal microbiota [48]. A healthy gut microbiome is characterized by high microbial diversity, which confers resilience against environmental stressors such as dietary shifts, infections, and antibiotic exposure. In contrast, gut dysbiosis disrupts immune regulation, increases systemic inflammation, and contributes to metabolic dysfunction [49](Table 1).

### Obesity

Obesity, defined by body mass index (BMI) greater than 30 kg/m² [50], is primarily driven by dietary and genetic factors, but is also increasingly recognized to be critically influenced by the gut microbiota [51]. Many studies have identified an increased *Firmicutes*-to-*Bacteroidetes* ratio in individuals with obesity [52]. For instance, studies have demonstrated that individuals with obesity exhibit higher abundances of bacterial genera such as *Porphyromonas*, *Campylobacter*, *Bacteroides*, *Staphylococcus*, *Parabacteroides*, *Dialister*, and *Ruminococcus* when compared to lean individuals [53]. On the other hand, the *Christensenellaceae* family, along with the genera *Methanobacteriales*, *Lactobacillus*, *Bifidobacteria*, and *Akkermansia*, are commonly recognized as probiotics, with their relative levels frequently showing an inverse correlation with obesity [54].

### Type 2 Diabetes (T2D)

T2D is a chronic metabolic disorder characterized by insulin resistance and insufficient insulin production, leading to prolonged hyperglycemia [55]. Gut microbiome studies in T2D patients have revealed a decline in the phylum *Firmicutes*, alongside an increase in the class *Betaproteobacteria* and the genus *Lactobacillus*, associated with elevated plasma glucose [56]. Additionally, there is a notable decrease in beneficial commensals in the phylum *Firmicutes* such as *Faecalibacterium prausnitzii* and *Roseburia intestinalis* and frequent depletion of other beneficial microbes, including genera *Bifidobacterium*, *Bacteroides*, and *Akkermansia* [57, 58]. These alterations are associated with microbial oxidative stress, β-cell dysfunction, and impaired glucose tolerance [57, 59]. This dysbiotic shift weakens gut barrier integrity, increasing gut permeability and LPS translocation, which causes metabolic endotoxemia, intensifies chronic inflammation, reduces SCFA production, and disrupts insulin signaling, exacerbating T2D complications [60].

### Inflammatory Bowel Disease (IBD)

IBD impacts around 1 million individuals in the United States, with a rising global prevalence, particularly in early adulthood [61]. IBD, which includes Crohn's disease (CD) and ulcerative colitis (UC), is linked with decreased microbial diversity and depletion of anti-inflammatory species such as *F. prausnitzii* and *Roseburia* [62, 63]. While the abundance of *Enterobacteria*, including species such as *E. coli*, has been shown to increase in IBD patients [64]. This dysbiosis exacerbates mucosal inflammation and weakens the epithelial barrier, leading to increased levels of pro-inflammatory cytokines such as tumor necrosis factor-α (TNF-α), IL-18 and IL-6 as well as reactive oxygen species (ROS), which further contribute to intestinal damage [65].

### The Respiratory Microbiome

The respiratory microbiome, encompassing microbial communities across the upper and lower airways, plays a critical role in maintaining pulmonary health and regulating immune responses [47]. Traditionally considered a sterile environment, the respiratory tract is now understood to host a dynamic and metabolically active microbiome, albeit with lower biomass and diversity compared to the gut microbiome [66]. The balance between commensal and pathogenic microorganisms across the respiratory tract is essential for immune equilibrium, protection against airborne pathogens, and preservation of pulmonary function [67] (Table 2).

### Asthma

Asthma is a chronic inflammatory airway disease marked by bronchial hyperresponsiveness, episodic airflow obstruction, and persistent inflammation [68]. The condition is frequently accompanied by microbial dysbiosis, typified by reduced microbial diversity and an overabundance of pathogenic taxa, which exacerbates airway inflammation and promotes disease progression [68]. This dysbiosis is characterized by reduced microbial diversity and overabundance of pathogenic taxa including *Proteobacteria* and *Firmicutes* at the phylum level. Within *Proteobacteria*, genera such as *Haemophilus*, *Moraxella*, and *Neisseria* are frequently overrepresented, and within *Firmicutes*, species such as *Streptococcus pneumoniae* and *Staphylococcus aureus* are enriched [69, 70]. Severe asthma is often associated with pathogenic colonization in the lower airways by *M.catarrhalis*, *H.influenzae*, and *Streptococcus* spp., triggering increased IL-5, IL-13, IL-8 and eosinophils [71, 72]. Conversely, the depletion of commensal bacteria such as those from the phyla *Firmicutes*, *Bacteroidetes*, and *Actinobacteria* compromises immune regulation and epithelial barrier integrity, further exacerbating the inflammatory milieu [73].

### Chronic Obstructive Pulmonary Disease (COPD)

COPD is a progressive and debilitating lung condition that ranks among the foremost contributors to the global disease burden, affecting over 300 million people worldwide [74]. In COPD, the lung microbiome undergoes significant alterations, with a notable loss of microbial diversity and a concurrent rise in pathogenic taxa. Predominant pathogens in COPD include *S. pneumoniae*, *S. aureus*, *M. catarrhalis*, *P. aeruginosa*, *Haemophilus* species and gram-negative enteric bacteria [75], which trigger neutrophilic inflammation, exacerbate symptoms, and accelerate lung damage. These bacteria form biofilms, enhancing persistent antibiotic resistance and impairing mucociliary clearance [76].

### Respiratory Infections

The viruses disrupt immune homeostasis and damage the respiratory epithelium, progressing to secondary bacterial infections, which worsen disease severity and delay recovery [77]. Common pathogens include *S. pneumoniae*, *S. aureus*, *M. catarrhalis*, *H. influenzae*, *K. pneumoniae*, and *P. aeruginosa* are frequently isolated in severe patients [78]. *S. pneumoniae* and *S. aureus* release cytotoxins, increasing epithelial apoptosis and bacterial proliferation [79], while *K. pneumoniae* and *M. catarrhalis* contribute to airway inflammation, especially in coinfections [80]. Beyond secondary infections, viral respiratory infections induce lung dysbiosis. Commensal bacteria, such as *Prevotella* and *Veillonella*, are frequently diminished [81], whereas pathogenic taxa from the *Proteobacteria* phylum, including *S. aureus*, *S. pneumoniae*, *H. influenzae*, and *M. catarrhalis* tend to be overrepresented [82], weakening epithelial barriers and promoting inflammation [83]. However, *Corynebacterium* and *Dolosigranulum* show a negative correlation with disease severity, highlighting their potential protective role [84].

### The Female Reproductive Tract Microbiome

The female reproductive tract microbiome is critical for immunity, reproduction, and infection resistance [85]. Once thought to be static, it is now recognized as highly dynamic, influenced by hormonal changes, age, lifestyle, and external factors [86]. In healthy women, *Lactobacillus* species including *L. jensenii*, *L. crispatus* and *L. gasseri*, secret lactic acid to maintain an acidic vaginal pH (3.5 to 4.5), [87] which inhibits the growth of pathogens, including *Gardnerella vaginalis* and *Candida albicans* [88]. Beyond acid production, *Lactobacillus* limits pathogen adherence through competitive exclusion, forms a protective biofilm, and enhances mucosal barrier integrity by stimulating epithelial tight junctions and producing immune-modulating cytokines [89]. Like other mucosal sites, dysbiosis in the vaginal microbiome disrupts the microbial balance and contributes to health issues [90](Table 3).

### Sexually Transmitted Infections (STI)

STIs are highly prevalent among sexually active individuals, with over 2.5 million cases reported in the United States [91]. *Chlamydia trachomatis* and *Neisseria gonorrhoeae* are common bacterial STIs and are often asymptomatic, leading to delayed diagnosis and complications such as infertility, and ectopic pregnancy [92, 93]. STI patients exhibit significant alterations in the vaginal microbiota, including reduced *Lactobacillus* levels and an increase in anaerobic bacteria such as *G. vaginalis*, *A. vaginae* [87, 94]. These microbial shifts raise vaginal pH and increase metabolites such as SCFAs, creating a favorable environment for pathogen colonization [95]. The loss of *Lactobacillus* reduces lactic acid and hydrogen peroxide production [96], weakening antimicrobial defenses and allowing *N. gonorrhoeae* and *C. trachomatis* to adhere to epithelial cells [97, 98]. Additionally, dysbiosis-associated anaerobes release inflammatory metabolites, stimulating the secretion of cytokines such as IL-1, IL-6, IL-12, and TNF-α. This exacerbates local inflammation, recruits immune cells, and weakens epithelial barriers, paradoxically facilitating pathogen survival and tissue damage [99]. The resulting inflammatory state sustains infection and increases susceptibility to other STIs, including human immunodeficiency virus (HIV) [100].

## The Functionality of NPs in the Mucosal System

### Organic NPs

**Protein-based NPs.** Protein-based NPs have emerged as a promising platform for modulating mucosal microbiota and immune responses due to their biocompatibility, biodegradability, and functional versatility [101]. These NPs are synthesized from natural or recombinant proteins, enabling the controlled delivery of bioactive compounds such as probiotics, antimicrobial peptides, and immunomodulatory agents [102]. Their ability to form stable nanostructures through self-assembly or engineered fabrication enhances their therapeutic potential in mucosal environments [101].

Protein-based NPs exert significant effects on the mucosal microbiota by selectively modulating microbial communities and enhancing probiotic viability. Encapsulation of *Lactobacillus* species in whey protein and zein protein NPs protects them from acidic degradation and improves their colonization efficiency within the GI tract [103]. Casein- and gelatin-based NPs have attracted considerable attention for their ability to protect probiotic viability and facilitate targeted delivery to the intestinal mucosa. These systems allow for efficient encapsulation and enable safe delivery without inducing cytotoxicity [104].

Beyond microbiota modulation, protein-based NPs influence mucosal immunity by enhancing the immune response and disease resistance as well as reinforcing epithelial barriers [105]. Albumin NPs loaded with bacterial lysates promote antigen presentation within mucosa-associated lymphoid tissue (MALT), leading to increased secretory immunoglobulin A (sIgA) production and enhanced mucosal immune responses [106]. Additionally, gelatin-based NPs have been shown to regulate inflammation by reducing pro-inflammatory cytokines such as TNF-α, IL-1 and IL-6 while promoting regulatory cytokines such as IL-10 and TGF-β [107, 108]. These immunomodulatory effects contribute to maintaining mucosal immune balance, which is critical in conditions such as IBD and allergic airway inflammation such as COPD [109, 110]. Moreover, protein-based NPs modulate mucosal barrier function by transiently opening epithelial tight junctions and interacting with goblet cells to influence mucin production, thereby facilitating drug transport while maintaining epithelial homeostasis [111].

The multifunctionality of protein-based NPs underscores their potential utility in mucosal-targeted applications. By modulating microbial composition, regulating local immune responses, and enhancing mucosal barrier integrity, these systems offer a promising framework for microbiome-centered therapeutic strategies. Nonetheless, further investigations are needed to refine their formulations, evaluate long-term safety, and establish reproducible clinical outcomes [112].

**Lipid-based NPs.** Lipid-based NPs are among the most established nanocarriers for mucosal delivery, owing to their biocompatibility, structural flexibility, and ability to encapsulate both hydrophilic and hydrophobic bioactive agents [113]. Common forms include liposomes, solid lipid nanoparticles (SLNs), nanostructured lipid carriers (NLCs), and lipid–polymer hybrid systems [114]. These systems are particularly suited for modulating mucosal microbiota and immune responses, as they can facilitate localized delivery to epithelial surfaces, enhance epithelial permeability, and protect labile compounds from enzymatic degradation in mucosal environments [115].

In mucosal systems, lipid-based NPs, such as curcumin-loaded liposomes combined with chitosan/gelatin multilayer coatings, have been used to enhance probiotic delivery. This strategy improves probiotic survival and adhesion while modulating gut microbiota—specifically increasing beneficial *Lactobacillus* and *Ruminococcaceae* and reducing inflammation-associated *Marinifilaceae* [116]. Lipid-based NPs contribute to mucosal immune modulation by facilitating the uptake of antigens and immunomodulators by APCs, such as DCs and macrophages [117]. Intranasal or oral mucosal delivery of lipid NPs has been shown to enhance local immune responses, including IgA secretion, and modulate T cell responses [118, 119].

At the epithelial barrier level, lipid-based NPs do not disrupt tight junction integrity, ensuring the preservation of mucosal barrier function. For example, in intestinal models, siRNA-loaded lipid NPs maintained transepithelial electrical resistance and the localization of tight junction proteins such as zonula occludens-1 (ZO-1), indicating no adverse effect on epithelial cohesion [120]. In contrast, pathological conditions such as IBD and colorectal cancer are associated with increased epithelial permeability due to tight junction disruption and immune cell infiltration. This so-called epithelial enhanced permeability and retention (EPR) effect facilitates the passive accumulation of NPs at inflamed sites, improving their potential for targeted delivery [121].

Collectively, these findings highlight the potential of lipid-based NPs as safe and efficient mucosal delivery platforms capable of modulating the microbiota, enhancing immune responses, and enabling targeted therapeutic action in both healthy and inflamed mucosal environments.

**Polysaccharide-based NPs.** Polysaccharide-based NPs have gained significant attention as mucosal delivery systems due to their biocompatibility, biodegradability, low immunogenicity, and intrinsic mucoadhesive properties [122]. In addition to these favorable characteristics, many of these NPs can selectively promote the growth of beneficial microbes, thereby modulating the gut environment. For instance, *Bifidobacterium* growth is promoted by octenyl succinic anhydride–modified starch NPs [123], and alginate hydrogel microspheres encapsulating *Bifidobacterium* enhance delivery to the colon and support proliferation while suppressing inflammation [124]. *Lactobacillus* populations are significantly enhanced by nanocrystalline cellulose [125], and alginate-based microspheres, which improve inflammatory gene expression, acid resistance, intestinal adhesion and SCFA expression [124].

In mucosal immunity, polysaccharide-based NPs influence immune responses by engaging pattern recognition receptors (PRRs), such as TLRs and C-type lectin receptors, found on mucosal DCs and macrophages [126]. Chitosan-based NPs have been shown to function as effective adjuvants by enhancing antigen uptake, promoting antigen presentation, and stimulating both humoral and cellular immune responses [126, 127].

**Fig. 2:**
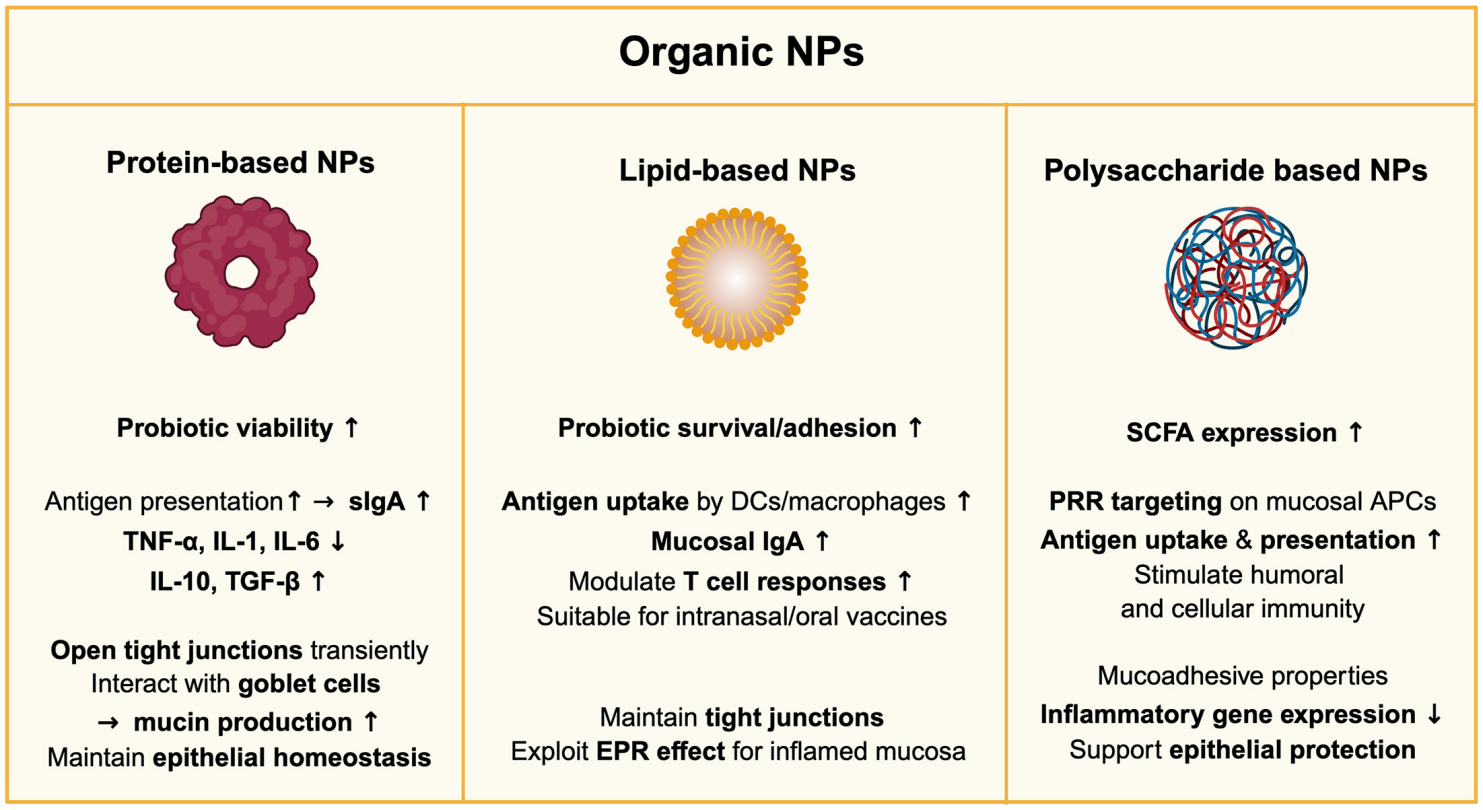
Functional properties of organic nanoparticles in the mucosal system. Key immunological and microbiological effects of protein-based, lipid-based, and polysaccharide-based nanoparticles at mucosal surfaces, including enhanced probiotic delivery, antigen uptake, immune modulation, and epithelial barrier support.

Overall, the versatility of polysaccharide-based NPs—encompassing microbial modulation, immune regulation, and epithelial protection—underscores their therapeutic potential in mucosal-targeted interventions. Future innovations in surface engineering, stimuli-responsive release systems, and combinatorial delivery of microbiome modulators will further strengthen their role in next-generation mucosal nanomedicine (Fig. 2).

### Inorganic NPs

**Titanium dioxide (TiO_2_) NPs.** TiO_2_ NPs are extensively utilized in diverse fields such as food processing, cosmetics, and pharmaceuticals due to their exceptional stability, biocompatibility, and photocatalytic properties [128]. These NPs have gained attention for their potential to influence the gut microbiome, particularly by targeting and eliminating pathogenic microorganisms that contribute to microbial imbalances [129].

TiO_2_ NPs exert their antimicrobial effects primarily by disrupting bacterial biofilms, which are complex extracellular matrices composed of polysaccharides, proteins, and DNA. These biofilms serve as protective barriers for various pathogens, including *P. aeruginosa*, *Proteus vulgaris*, *Acinetobacter baumannii*, *Serratia marcescens*, and *E. coli* [130, 131]. Furthermore, TiO_2_ NPs show the capability to generate ROS under physiological conditions such as neutral pH and body temperature. This ROS production effectively degrades key biofilm components including polysaccharides, proteins, and extracellular DNA, thereby compromising biofilm structural integrity and increasing pathogen susceptibility [132]. Furthermore, TiO_2_ NPs have been reported to influence the mucus layer within the gut, modulating its thickness and composition [133]. These effects, particularly on acidic and neutral mucins, may enhance mucosal defenses by reducing pathogen adherence and invasion, thereby bolstering the intestinal barrier against microbial stressors [134].

Despite their potential, the application of TiO_2_ NPs in gut microbiome modulation remains controversial due to challenges related to their long-term safety, potential bioaccumulation, and unintended effects on microbial communities [135]. For instance, experimental studies have indicated that exposure to TiO_2_ NPs can inadvertently disrupt beneficial microbial populations such as *Lactobacillus* [136]. Additionally, TiO_2_ NPs have been observed to stimulate host cells to produce pro-inflammatory cytokines, including IL-6 and IL-8, which may exacerbate gut inflammation under certain conditions such as high NP concentrations in the presence of pre-dispersed formulations, or depending on specific physicochemical properties such as surface area, crystallinity, and dispersion state [137, 138]. This highlights the need for careful nanoparticle design to minimize unintended immune responses in both gut and lung microbiomes.

Such findings emphasize the need for surface modifications and dosage optimization of TiO_2_ NPs to balance their antimicrobial benefits while minimizing inflammatory and microbial disruption risks [139]. Moreover, evidence from oral toxicity studies comparing surface-treated and untreated TiO_2_ particles indicates no adverse impact on toxicity, even at very high doses, highlighting the importance of carefully designing nanoparticle interventions [140]. Further research is essential to elucidate the long-term interactions between TiO_2_ NPs, host immune responses, and the gut microbiome.

**Silver (Ag) NPs.** Ag NPs have emerged as a potent antimicrobial agent, with significant efficacy against a wide range of pathogenic microorganisms, including multidrug-resistant strains [141]. Their antimicrobial properties are largely attributed to the generation of ROS, which induce oxidative stress and cause structural disruption in the bacterial membrane, such as membrane perforation or pore formation [142]. In addition to inducing the generation of ROS, the toxicity of Ag NPs compounds arises mainly from the release of ions that compromise the cell envelope’s integrity by destabilizing the membrane [143]. These ions also interact with nucleic acids and proteins, interfering with replication and synthesis processes [144], and inhibiting essential metabolic pathways [145]. Additionally, by dismantling biofilm structures, Ag NPs effectively enhance the susceptibility of embedded bacteria to antimicrobial agents, thereby addressing one of the most challenging aspects of infection management [146].

Although Ag NPs demonstrate considerable antimicrobial potential, their application presents notable challenges and limitations. While they exhibit antimicrobial effects under certain conditions, Ag NPs may decrease the *Firmicutes*/*Bacteroidetes* ratio, including *Lactobacillus* [147]. Notably, silver and its compounds are broadly effective against both Gram-positive and Gram-negative bacteria [148]. However, some *in vivo* findings suggest that Ag NP exposure can shift gut microbiota composition toward greater proportions of Gram-negative bacteria, whereas others report no significant alterations in these bacterial phyla [149].

Additionally, the interaction of Ag NPs with host cells presents both opportunities and challenges. On one hand, their ability to modulate inflammatory responses, including the reduction of pro-inflammatory cytokines, offers therapeutic potential for conditions characterized by excessive gut inflammation [150]. On the other hand, Ag NPs induce the release of cytokines such as TNF-α, macrophage inhibitory protein (MIP-2) and IL-1β in a size-dependent manner, potentially exacerbating inflammation [151]. However, recent evidence suggests that prophylactic administration of Ag NPs to the lungs can reduce viral loads and virus-induced cytokines, partly by recruiting and regulating lymphoid cells, including NK cells, activated through their interaction with alveolar macrophages [152]. These dual actions underscore the importance of a comprehensive understanding of how Ag NPs interact with both the microbiota and the host immune system.

The long-term safety of Ag NPs remains an area of active research. Concerns about bioaccumulation and the potential for inducing microbial resistance necessitate comprehensive toxicological evaluations. An *in vitro* study revealed that prolonged exposure of BEAS-2B cells to Ag NPs leads to the upregulation of *TGFβ1* and promotes epithelial-mesenchymal transition and cellular transformation, as demonstrated by RNA sequencing analysis [153].

**Fig. 3:**
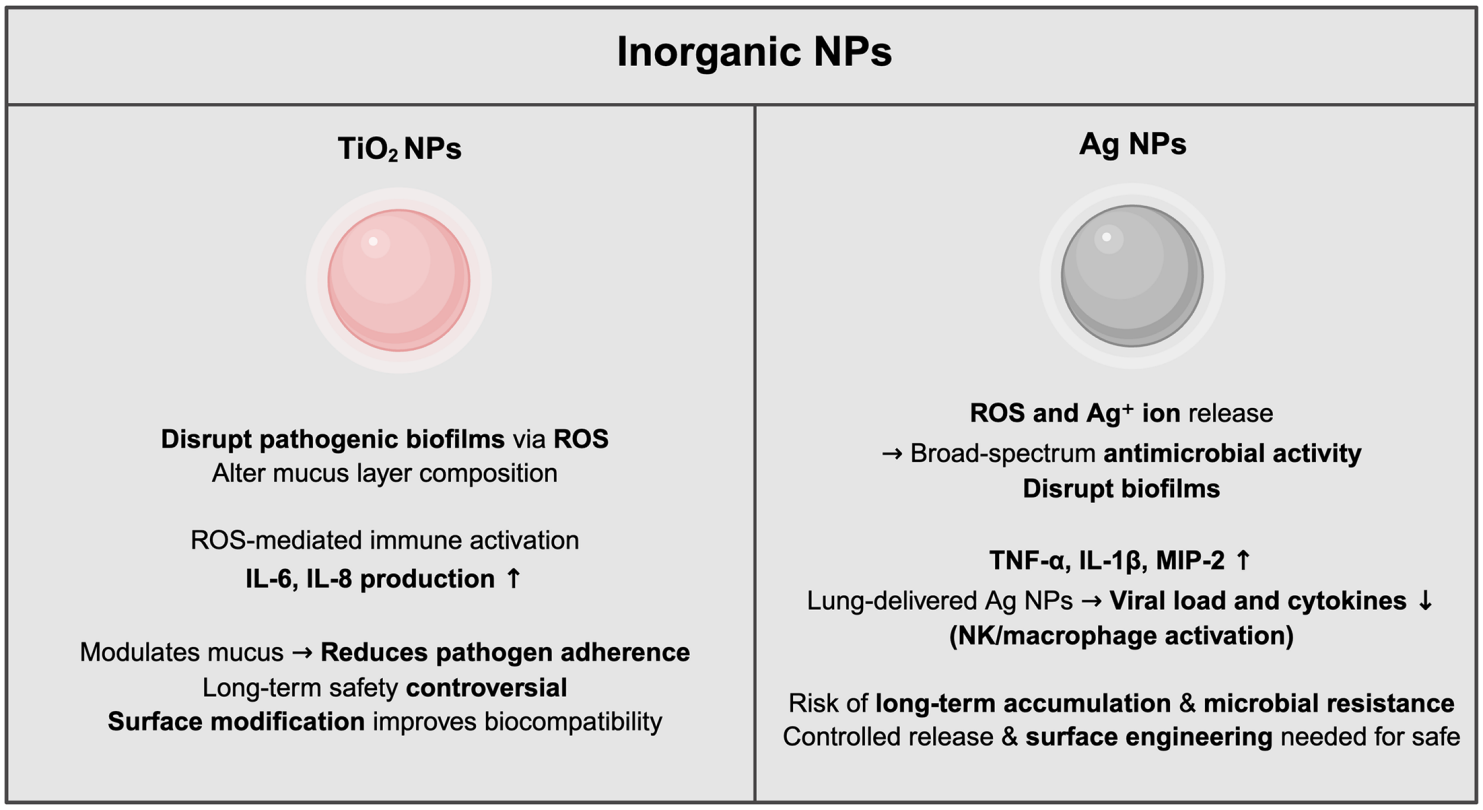
Functional properties of inorganic nanoparticles in the mucosal system. Immunologically and microbiologically relevant actions of titanium dioxide (TiO_2_) and silver (Ag) nanoparticles at mucosal surfaces, including ROS-driven antimicrobial activity, biofilm disruption, regulation of immune signaling, and modulation of mucus characteristics and microbial community structure.

Although emerging evidence supports their potential for gut microbiome modulation, achieving an optimal balance between therapeutic efficacy and safety remains essential. Advances in nanoparticle engineering, particularly through surface functionalization and controlled release mechanisms, hold the potential to address these challenges and unlock the full therapeutic potential of Ag NPs [154] (Fig. 3).

## Application of NPs as a Direct Modulator of Mucosal Microbiota Dynamics

Probiotics, which are live microorganisms that provide health benefits to the host when consumed in sufficient quantities, and prebiotics, which are indigestible dietary components that selectively enhance the growth and activity of beneficial microorganisms in the GI tract, have gained significant interest in the areas of functional foods, nutraceuticals, and therapeutic applications [155]. Both probiotics and prebiotics play pivotal roles in maintaining gut health, modulating immune responses, and preventing dysbiosis—a microbial imbalance associated with various health conditions [156]. However, conventional formulations of probiotics and prebiotics often face significant limitations, including low survival rates during storage and transit through the harsh acidic environment of the stomach, inadequate colonization in the host’s gut, and premature degradation or nonspecific utilization of prebiotics before reaching their target sites [157]. The integration of nanoparticle-based delivery systems into probiotics and prebiotics research has introduced a transformative approach to addressing dysbiosis across diverse mucosal environments. Probiotic and prebiotic NPs leverage nanotechnology to overcome inherent limitations such as low stability, inefficient delivery, and limited therapeutic efficacy in conventional formulations [158].

The encapsulation techniques enhance survival, target site specificity, and bioavailability, paving the way for innovative strategies to restore microbial balance and support host health [159]. Various encapsulation strategies have been employed to optimize delivery efficiency. Polysaccharide-based encapsulation, using materials such as alginate, chitosan, and pectin, provides a protective matrix that enhances probiotic survival under harsh gastric conditions while enabling controlled intestinal release and low immunogenicity [160-162]. Protein-based encapsulation, utilizing carriers such as whey protein, casein, and gelatin, offers structural stability and gradual degradation, making it suitable for sustained probiotic and prebiotic release [163-165]. Lipid-based encapsulation, such as liposomes and solid lipid NPs, enhances probiotic efficiency while providing a protective barrier against gastric conditions, but is prone to oxidation and thermal instability [158, 166]. Polymer-based encapsulation, employing biocompatible materials such as hydrogels, chitosan derivatives, and gelatin scaffolds, enables enhanced protection and stimuli-responsive targeted release [167, 168].

Probiotics can modify gut microbiota by promoting the production of SCFAs and lactic acid, while also enhancing the generation of AMPs such as lactobin A, curvacin A, enterocin, and pediocin [169-173]. Several probiotic strains, including *Lactobacillus* spp. (*L. acidophilus*, *L. amylovorus*, *L. brevis*, *L. bulgaricus*, *L. casei*, *L. curvatus*, *L. helveticus*, *L. lactis*, and *L. plantarum*), *Leuconostoc gelidum*, *Enterococcus faecium* (*E. faecium* CTC492, *E. faecium* T136, and *E. faecium* P13), and *Pediococcus* spp. (*P. acidilactici* and *P. pentosaceus*), are well known for their ability to produce these AMPs, contributing to gut homeostasis and pathogen inhibition [174]. Additionally, these systems regulate the activity of DCs, T cells, and B cells, suppressing inflammatory responses and modulating the immune system [175, 176]. On the other hand, prebiotic NPs encapsulate substrates such as inulin, fructooligosaccharides (FOS), and galactooligosaccharides (GOS) within nano-size carriers made of biocompatible materials [177-179]. Inulin-based NPs effectively regulate gut microbiota, enhance SCFA production, and improve anti-inflammatory immune responses, showing potential in colorectal cancer therapy by increasing regorafenib accumulation in tumors and polarizing tumor-associated macrophages toward an M1 phenotype [180]. Additionally, GOS-loaded PLGA NPs have been shown to enhance gut barrier integrity, promote SCFA production, and activate T-regulatory cells, thereby improving gut permeability and modulating gut-associated immune responses in models of intestinal inflammation [181].

The co-encapsulation of probiotics and prebiotics into a single nanoparticle delivery system introduces the concept of synbiotic NPs. These systems enable simultaneous and localized delivery of both components, amplifying their synergistic effects [182]. For instance, prebiotics such as chitosan, protein, cellulose and inulin within the nanoparticles act as a nutrient source for co-delivered probiotics such as *Lactobacillus*, enhancing their colonization and metabolic activity and overall therapeutic efficacy [183-187]. In the Drosophila model, *Lactobacillus fermentum* was encapsulated with chitosan, a prebiotic, to create a synbiotic nanoparticle system. This approach not only stabilized the probiotics during gut transit but also enhanced their immunomodulatory effects, mitigating acrylamide-induced toxicity by modulating gut microbiota and reducing oxidative stress [184]. This integrated approach maximizes SCFA production, strengthens mucosal defenses, and accelerates the restoration of microbial equilibrium. Encapsulating probiotics such as *Bacillus amyloliquefaciens*, *L. acidophilus*, and *Bifidobacterium bifidum* in chitosan NPs improves their survival in acidic and intestinal conditions, effectively delivering them to the colon [188]. This encapsulation reduces inflammation, enhances anti-inflammatory cytokine expression, and restores epithelial integrity in colitis models, highlighting their utility in treating IBD [189].

Furthermore, other advanced nanoparticle formulations have demonstrated notable benefits. Chitosan-coated PLGA NPs loaded with lyophilized probiotic extract have demonstrated targeted delivery to inflamed colon tissues, significantly reducing pro-inflammatory cytokines, lipid peroxidation, and myeloperoxidase activity, as well as improving colonic histopathological conditions in murine colitis models [187]. Additionally, lipid NP based delivery systems, such as SLNs loaded with rosiglitazone and probiotics, enhance the stability and viability of probiotics, offering antioxidant activity and sustained release properties, and demonstrating potential for diverse applications [190]. Ag and TiO_2_ NPs exhibit antimicrobial activity against beneficial bacteria such as *L. casei*, *L. plantarum*, and *L. fermentum*. However, the presence of prebiotics such as raffinose, lactulose, and inulin significantly mitigates their decline, suggesting a protective role of prebiotics in buffering the antimicrobial effects of NPs and supporting the survival of beneficial bacteria [191]. Additionally, studies on Ag NP exposure in gut microbiota models indicate that while core bacterial communities remain stable, rare species exhibit significant fluctuations, and *Firmicutes* to *Bacteroidetes* ratios shift. The co-administration of probiotics such as *Bacillus subtilis* alleviated these effects by maintaining microbial homeostasis and preventing metabolic disruptions [192]. Similarly, TiO_2_ NP exposure disrupts gut microbiota, depletes *Lactobacillus*, and induces colonic inflammation via NF-κB activation. While *Lactobacillus rhamnosus* GG offers protective effects, further research is needed to assess long-term risks and refine probiotic interventions [193].

**Fig. 4:**
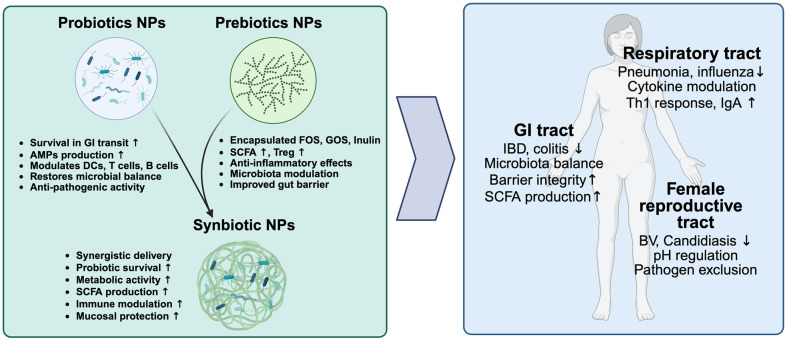
Functions and mucosal effects of probiotic, prebiotic, and synbiotic NPs. (Left) Functions of each NP type. Probiotic NPs enhance gastrointestinal survival, immune modulation, and microbial balance. Prebiotic NPs promote shortchain fatty acid (SCFA) production, anti-inflammatory activity, and gut barrier function. Synbiotic NPs co-deliver both components, resulting in synergistic effects, including improved colonization, metabolism, and mucosal protection. (Right) Combined mucosal effects. In the GI tract, NPs reduce inflammation, restore microbiota balance, enhance barrier integrity, and increase SCFA levels. In the respiratory tract, they modulate immune responses and enhance IgA production. In the female reproductive tract, they contribute to infection prevention, pH regulation, and pathogen exclusion.

In the respiratory tract, various strategies using probiotic, prebiotic and synbiotic NPs offer an innovative approach to enhance the effectiveness of respiratory treatments. *L. rhamnosus* encapsulated in oleic acid–substituted chitosan-linoleic acid–retinol (OASCLR) NPs enable targeted respiratory delivery via intratracheal administration, enhancing stability and probiotic activity. These NPs facilitated the macrophage transition from M1 to M2 through CD44-hyaluronic acid (HA) interaction, mitigating excessive immune response in bacterial pneumonia. Additionally, OASCLR NPs modulate inflammation by reducing TNF-α and increasing IL-10 levels [194]. In the female reproductive tract, maintaining a *Lactobacillus*-dominant microbiome is crucial for preventing infections such as BV and candidiasis. HA hydrogel offers an innovative solution by ensuring the delivery and survival of *Lactobacillus* species, which produce lactic acid and hydrogen peroxide to inhibit pathogens such as *G. vaginalis* and *C. albicans* [195, 196]. In addition, *L. rhamnosus* and *L. gasseri* immobilized in electrospun polymeric nanofibers exhibited potent inhibitory effects against pathogens such as *G. vaginalis* and *C. albicans*, while maintaining high survival rates and stability during long-term storage, suggesting a promising strategy for enhancing vaginal health through innovative delivery systems [197]. However, despite these promising findings, the continuous turnover of vaginal mucus poses a significant challenge to the retention and effectiveness of NPs potentially limiting their therapeutic impact [198, 199]. Thus, further research is needed to provide consistent and robust evidence supporting the efficacy of these interventions [200] (Fig. 4).

## Conclusion and Future Directions

**Fig. 5:**
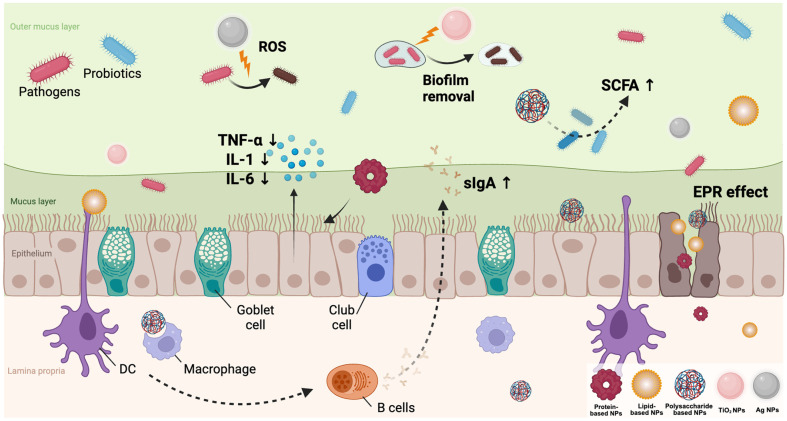
Overview of nanoparticle-mediated modulation of the mucosal immune system and microbiota. Within the mucosal environment, NPs enhance probiotic viability, suppress pro-inflammatory cytokines (*e.g.*, TNF-α, IL-1, IL-6), promote sIgA production, maintain epithelial barrier integrity, and modulate local immune cell activity. NPs can accumulate at inflamed sites via the enhanced permeability and retention (EPR) effect, thereby facilitating targeted therapeutic delivery.

NPs have emerged as transformative tools in the modulation of mucosal immunity and microbiome dynamics. This review highlights the intricate interplay between the immune system, microbial ecosystems, and nanoparticle technologies within the mucosal environment. NPs influence the mucosal immune response both directly through interactions with epithelial and immune cells, and indirectly by modulating the composition and metabolic activity of microbiota. Such modulation of the microbiome plays a crucial role in restoring the balance between innate and adaptive immune responses, maintaining immune tolerance, and alleviating inflammation. Consequently, NP-mediated microbiome regulation holds substantial potential for the prevention and treatment of various mucosal-associated diseases, including IBD, airway disorders, and infectious diseases. By leveraging these interactions, NPs demonstrate significant potential in restoring microbial homeostasis and mitigating inflammation. Notably, NPs offer promising strategies for addressing dysbiosis through precise modifications of the mucosal microbiome, thereby contributing to the treatment of related disorders (Fig. 5).

However, several critical limitations must be addressed to fully realize this potential. First, while the short-term efficacy of nanoparticle-based therapies has been well-documented in preclinical studies, data on their long-term safety, effectiveness, and potential adverse effects remain limited. The long-term interactions between NPs and host-microbial communities, as well as the immune system, are not yet fully understood, underscoring the need for comprehensive and longitudinal studies. Furthermore, although preclinical research has provided valuable insights, clinical trials involving human subjects remain sparse. Robust, large-scale, and long-term clinical trials are essential to validate the safety and efficacy of nanoparticle-based interventions and establish their translational viability.

Additionally, the risk of off-target effects represents a significant challenge in the application of nanoparticle technologies. Despite their high specificity, NPs may inadvertently interact with commensal microorganisms or host tissues, potentially disrupting microbial homeostasis or impairing immune regulation. Such unintended effects could diminish therapeutic outcomes or even exacerbate existing conditions. To mitigate these risks, advancements in nanoparticle design and functionalization are required to enhance their selectivity and minimize off-target interactions. In this context, some NPs hold particular promise for probiotic delivery. By promoting survival and targeted delivery of beneficial microorganisms, these systems can stabilize the mucosal environment and restore microbial balance, thereby amplifying therapeutic efficacy.

In conclusion, NPs represent a powerful and versatile platform for the modulation of mucosal immunity and the microbiome. However, addressing key limitations, such as the lack of long-term data, limited clinical trials, and the potential for off-target effects, is critical for advancing this field. Overcoming these challenges will pave the way for NPs to serve as innovative solutions in the treatment of mucosal immune-related disorders and microbiome-associated diseases.

## Figures and Tables

**Table 1:** Diseases associated with gut microbiome dysbiosis.

Disease	Microbiome change	Functional effect	Ref.
Obesity	*Firmicutes*/*Bacteroidetes* ratio ↑	Enhanced carbohydrate fermentation and nutrient absorption	[52]
	*Porphyromonas*, *Campylobacter*, *Bacteroides*, *Staphylococcus*, *Parabacteroides*, *Dialister*, *Ruminococcus* ↑	Associated with low microbial diversity, inflammation, and metabolic dysregulation	[53]
	*Christensenellaceae*, *Methanobacteriales*, *Bifidobacteria*, *Akkermansia* ↓	Reduced probiotics linked to inflammation and metabolic dysfunction	[54]
Type 2 Diabetes (T2D)	*Firmicutes* ↓ *Betaproteobacteria*, *Lactobacillus* ↑	Reduced glucose tolerance, systemic inflammation, and metabolic dysregulation	[56]
	*F. prausnitzii*, *R. intestinalis* ↓	Loss of butyrate producers linked to reduced insulin sensitivity and impaired glucose regulation	[57]
	*Bifidobacterium*, *Bacteroides*, *Akkermansia* ↓	Impaired gut barrier, immune dysfunction, and reduced insulin sensitivity	[58]
Inflammatory Bowel Disease (IBD)	*F. prausnitzii*, *Bifidobacterium*, *Firmicutes* ↓	Reduced butyrate production, impaired mucosal protection, and increased inflammation	[62]
	*Clostridium leptum* group, *Firmicutes* ↓	Reduced butyrate production and impaired mucosal immune regulation	[63]
	*Bacteroides* group, *Bifidobacterium* spp., *Clostridium leptum group*, *Firmicutes* ↓ *Enterobacteria* ↑	Pro-inflammatory potential and epithelial adhesion	[64]

**Table 2:** Diseases associated with respiratory microbiome dysbiosis.

Disease	Microbiome Change	Functional Effect	Ref.
Asthma	*Bacteroides* spp. ↓ *Haemophilus*, *Moraxella*, *Streptococcus*, *Staphylococcus* ↑	Loss of immune regulation, steroid resistance, neutrophilic inflammation, and increased risk of exacerbation	[69]
	*Lactobacillales*, *Mogibacteriaceae*, *Veillonella*, *Prevotella* ↓ *M. catarrhalis*, *H. influenzae*, *Streptococcus* spp. ↑	Neutrophilic inflammation, steroid resistance, impaired immune regulation (IL-5, IL-6, IL-13, IL-8, IL-17A ↑ )	[71, 72]
	*Firmicutes*, *Bacteroidetes*, *Actinobacteria* ↓ *Proteobacteria* ↑	Reduced microbial diversity, impaired lung function, and a shift toward a proinflammatory microbial profile	[73]
COPD	*Prevotella*, *Veillonella*, *Gemella* ↓ *Haemophilus*, *Moraxella*, *S. pneumoniae*, *S. aureus*, *P. aeruginosa*, gram-negative enteric bacteria ↑	Neutrophilic inflammation, reduced lung function, immune exhaustion, and decreased microbial diversity (IL-8, TNF, IL-1β ↑ )	[75]
	*Prevotella*, *Veillonella* ↓ *H. influenzae*, *S. pneumoniae*, *M. catarrhalis* ↑	Biofilm-mediated immune evasion, persistent neutrophilic inflammation, and reduced antibiotic responsiveness	[76]
Respiratory Infections	*Prevotella*, *Veillonella*, *Rothia* spp. ↓ *H. influenzae*, *S. pneumoniae*, *M. catarrhalis* ↑	Reduced diversity, immune imbalance, and increased susceptibility to symptomatic viral infection	[81]
	*Prevotella*, *Veillonella* ↓ *H. influenzae*, *M. catarrhalis*, *S. pneumoniae* ↑	Impaired mucosal immunity, increased susceptibility to viral and bacterial coinfection	[82, 83]
	*Corynebacterium*, *Dolosigranulum* ↓	Negative correlation with disease severity, IFN-γ, IL-33 ↓	[84]

**Table 3:** Diseases associated with female reproductive microbiome dysbiosis.

Disease	Microbiome Change	Functional Effect	Ref.
STI	*Lactobacillus* spp. ↓ BV-associated anaerobes (*G. vaginalis*, *Prevotella*) ↑	Reduced mucosal defense, Risk of asymptomatic STIs and complications (infertility, ectopic pregnancy)	[92, 93]
	*Lactobacillus* spp. ↓ BV-associated anaerobes (*G. vaginalis*, *A. vaginae*, *Prevotella* spp.) ↑	Increased vaginal pH, reduced lactic acid, elevated SCFAs, impaired barrier, increased proinflammatory cytokines	[94, 95]
	*Lactobacillus* spp. ↓ BV-associated anaerobes (*G. vaginalis*, *A. vaginae*, *Prevotella*, Megasphaera) ↑	Reduced lactic acid and H_2_O_2_; increased pH and SCFAs; elevated IL-1β, IL-6, IL-8; epithelial barrier disruption; enhanced STI pathogen adhesion and invasion	[97-99]

## References

[1] Kaiser P (1984). Physical performance and muscle metabolism during beta-adrenergic blockade in man. Acta Physiol. Scand. Suppl..

[2] Ozcam M, Lynch SV (2024). The gut-airway microbiome axis in health and respiratory diseases. Nat. Rev. Microbiol..

[3] From the American Association of Neurological Surgeons ASoNC, Interventional Radiology Society of Europe CIRACoNSESoMINTESoNESOSfCA, Interventions SoIRSoNS, World Stroke O, Sacks D, Baxter B, *et al*. 2018. Multisociety consensus quality improvement revised consensus statement for endovascular therapy of acute ischemic stroke. *Int. J. Stroke* **13:** 612-632. 10.1177/1747493018778713 29786478

[4] Takiishi T, Fenero CIM, Camara NOS (2017). Intestinal barrier and gut microbiota: shaping our immune responses throughout life. Tissue Barriers.

[5] Sardinha-Silva A, Alves-Ferreira EVC, Grigg ME (2022). Intestinal immune responses to commensal and pathogenic protozoa. Front. Immunol..

[6] De Martinis M, Sirufo MM, Suppa M, Ginaldi L (2020). New perspectives in food allergy. Int. J. Mol. Sci..

[7] Neish AS (2014). Mucosal immunity and the microbiome. Ann. Am. Thorac. Soc..

[8] Celebi Sozener Z, Ozdel Ozturk B, Cerci P, Turk M, Gorgulu Akin B, Akdis M (2022). Epithelial barrier hypothesis: effect of the external exposome on the microbiome and epithelial barriers in allergic disease. Allergy.

[9] Chen X, Lu Y, Chen T, Li R (2021). The female vaginal microbiome in health and bacterial vaginosis. Front. Cell. Infect. Microbiol..

[10] Ihekweazu FD, Versalovic J (2018). Development of the pediatric gut microbiome: impact on health and disease. Am. J. Med. Sci..

[11] McCright J, Ramirez A, Amosu M, Sinha A, Bogseth A, Maisel K (2021). Targeting the gut mucosal immune system using nanomaterials. Pharmaceutics.

[12] Chuaqui R, Verni J (1993). [Diagnosis of human papillomavirus infections in cervical cytology in the absence of classical signs]. Rev. Chil. Obstet. Ginecol..

[13] Lin S, Mukherjee S, Li J, Hou W, Pan C, Liu J (2021). Mucosal immunity-mediated modulation of the gut microbiome by oral delivery of probiotics into Peyer's patches. Sci. Adv..

[14] Scaldaferri F, Pizzoferrato M, Gerardi V, Lopetuso L, Gasbarrini A. 2012. The gut barrier: new acquisitions and therapeutic approaches. *J. Clin. Gastroenterol.* **46 Suppl:** S12-17. 10.1097/MCG.0b013e31826ae849 22955350

[15] Mulet-Powell N, Lacoste-Armynot AM, Vinas M, Simeon de Buochberg M (1998). Interactions between pairs of bacteriocins from lactic bacteria. J. Food Prot..

[16] Monin L, Whettlock EM, Male V (2020). Immune responses in the human female reproductive tract. Immunology.

[17] Yoo JS, Oh SF (2023). Unconventional immune cells in the gut mucosal barrier: regulation by symbiotic microbiota. Exp. Mol. Med..

[18] Ayres JS (2016). Cooperative Microbial tolerance behaviors in host-microbiota mutualism. Cell.

[19] Buckley A, Turner JR (2018). Cell biology of tight junction barrier regulation and mucosal disease. Cold Spring Harb. Perspect. Biol..

[20] Soderholm AT, Pedicord VA (2019). Intestinal epithelial cells: at the interface of the microbiota and mucosal immunity. Immunology.

[21] Ranjbar R, Vahdati SN, Tavakoli S, Khodaie R, Behboudi H (2021). Immunomodulatory roles of microbiota-derived short-chain fatty acids in bacterial infections. Biomed. Pharmacother..

[22] Kim J, Cho BH, Jang YS (2023). Understanding the roles of host defense peptides in immune modulation: from antimicrobial action to potential as adjuvants. J. Microbiol. Biotechnol..

[23] Didriksen BJ, Eshleman EM, Alenghat T (2024). Epithelial regulation of microbiota-immune cell dynamics. Mucosal. Immunol..

[24] Zheng M, Sun S, Zhou J, Liu M (2021). Virulence factors impair epithelial junctions during bacterial infection. J. Clin. Lab. Anal..

[25] Bricault I, Ferrettio G, Cinquin P (1995). Computer-assisted bronchoscopy: aims and research perspectives. J. Image Guid. Surg..

[26] Wanford JJ, Hachani A, Odendall C (2022). Reprogramming of cell death pathways by bacterial effectors as a widespread virulence strategy. Infect. Immun..

[27] Wershil BK, Furuta GT (2008). 4. Gastrointestinal mucosal immunity. J. Allergy Clin. Immunol..

[28] Wynn TA, Vannella KM (2016). Macrophages in tissue repair, regeneration, and fibrosis. Immunity.

[29] Cicchese JM, Evans S, Hult C, Joslyn LR, Wessler T, Millar JA (2018). Dynamic balance of pro- and anti-inflammatory signals controls disease and limits pathology. Immunol. Rev..

[30] Morhardt TL, Hayashi A, Ochi T, Quiros M, Kitamoto S, Nagao-Kitamoto H (2019). IL-10 produced by macrophages regulates epithelial integrity in the small intestine. Sci. Rep..

[31] Fernandez S, Jose P, Avdiushko MG, Kaplan AM, Cohen DA (2004). Inhibition of IL-10 receptor function in alveolar macrophages by Toll-like receptor agonists. J. Immunol..

[32] Doan TA, Forward T, Tamburini BAJ (2022). Trafficking and retention of protein antigens across systems and immune cell types. Cell. Mol. Life Sci..

[33] Chistiakov DA, Bobryshev YV, Kozarov E, Sobenin IA, Orekhov AN (2014). Intestinal mucosal tolerance and impact of gut microbiota to mucosal tolerance. Front. Microbiol..

[34] Guo N, Lv LL (2023). Mechanistic insights into the role of probiotics in modulating immune cells in ulcerative colitis. Immun. Inflamm. Dis..

[35] Smith NM, Wasserman GA, Coleman FT, Hilliard KL, Yamamoto K, Lipsitz E (2018). Regionally compartmentalized resident memory T cells mediate naturally acquired protection against pneumococcal pneumonia. Mucosal. Immunol..

[36] Al-Qahtani AA, Alhamlan FS, Al-Qahtani AA (2024). Pro-inflammatory and anti-inflammatory interleukins in infectious diseases: a comprehensive review. Trop. Med. Infect. Dis..

[37] van Wijk F, Cheroutre H (2010). Mucosal T cells in gut homeostasis and inflammation. Expert Rev. Clin. Immunol..

[38] Turner DL, Farber DL (2014). Mucosal resident memory CD4 T cells in protection and immunopathology. Front. Immunol..

[39] Luckheeram RV, Zhou R, Verma AD, Xia B (2012). CD4(+)T cells: differentiation and functions. Clin. Dev. Immunol..

[40] Galeano Nino JL, Pageon SV, Tay SS, Colakoglu F, Kempe D, Hywood J (2020). Cytotoxic T cells swarm by homotypic chemokine signalling. Elife.

[41] Dwivedy A, Aich P (2011). Importance of innate mucosal immunity and the promises it holds. Int. J. Gen. Med..

[42] Di Tommaso N, Gasbarrini A, Ponziani FR (2021). Intestinal barrier in human health and disease. Int. J. Environ. Res. Public Health.

[43] Robinson CJ, Bohannan BJ, Young VB (2010). From structure to function: the ecology of host-associated microbial communities. Microbiol. Mol. Biol. Rev..

[44] Schloss PD, Handelsman J (2005). Metagenomics for studying unculturable microorganisms: cutting the Gordian knot. Genome Biol..

[45] Kennedy MS, Chang EB (2020). The microbiome: composition and locations. Prog. Mol. Biol. Transl. Sci..

[46] Moszak M, Szulinska M, Bogdanski P (2020). You are what you eat-the relationship between diet, microbiota, and metabolic disorders-A review. Nutrients.

[47] Hou K, Wu ZX, Chen XY, Wang JQ, Zhang D, Xiao C (2022). Microbiota in health and diseases. Signal. Transduct. Target. Ther..

[48] Anto L, Blesso CN (2022). Interplay between diet, the gut microbiome, and atherosclerosis: role of dysbiosis and microbial metabolites on inflammation and disordered lipid metabolism. J. Nutr. Biochem..

[49] Talapko J, Vcev A, Mestrovic T, Pustijanac E, Jukic M, Skrlec I (2022). Homeostasis and dysbiosis of the intestinal microbiota: comparing hallmarks of a healthy state with changes in inflammatory bowel disease. Microorganisms.

[50] Okunogbe A, Nugent R, Spencer G, Powis J, Ralston J, Wilding J (2022). Economic impacts of overweight and obesity: current and future estimates for 161 countries. BMJ Glob. Health.

[51] Boulange CL, Neves AL, Chilloux J, Nicholson JK, Dumas ME (2016). Impact of the gut microbiota on inflammation, obesity, and metabolic disease. Genome Med..

[52] Palmas V, Pisanu S, Madau V, Casula E, Deledda A, Cusano R (2021). Gut microbiota markers associated with obesity and overweight in Italian adults. Sci. Rep..

[53] Le Chatelier E, Nielsen T, Qin J, Prifti E, Hildebrand F, Falony G (2013). Richness of human gut microbiome correlates with metabolic markers. Nature.

[54] Liu BN, Liu XT, Liang ZH, Wang JH (2021). Gut microbiota in obesity. World J. Gastroenterol..

[55] Iatcu CO, Steen A, Covasa M (2021). Gut microbiota and complications of type-2 diabetes. Nutrients.

[56] Larsen N, Vogensen FK, van den Berg FW, Nielsen DS, Andreasen AS, Pedersen BK (2010). Gut microbiota in human adults with type 2 diabetes differs from non-diabetic adults. PLoS One.

[57] Karlsson FH, Tremaroli V, Nookaew I, Bergstrom G, Behre CJ, Fagerberg B (2013). Gut metagenome in European women with normal, impaired and diabetic glucose control. Nature.

[58] Gurung M, Li Z, You H, Rodrigues R, Jump DB, Morgun A (2020). Role of gut microbiota in type 2 diabetes pathophysiology. EBioMedicine.

[59] Wright E, Jr., Scism-Bacon JL, Glass LC. 2006. Oxidative stress in type 2 diabetes: the role of fasting and postprandial glycaemia. *Int. J. Clin. Pract.* **60:** 308-314. 10.1111/j.1368-5031.2006.00825.x 16494646 PMC1448694

[60] Sircana A, Framarin L, Leone N, Berrutti M, Castellino F, Parente R (2018). Altered gut microbiota in type 2 diabetes: just a coincidence?. Curr. Diab. Rep..

[61] Kaplan GG (2015). The global burden of IBD: from 2015 to 2025. Nat. Rev. Gastroenterol. Hepatol..

[62] Sokol H, Seksik P, Furet JP, Firmesse O, Nion-Larmurier I, Beaugerie L (2009). Low counts of *Faecalibacterium prausnitzii* in colitis microbiota. Inflamm. Bowel Dis..

[63] Manichanh C, Rigottier-Gois L, Bonnaud E, Gloux K, Pelletier E, Frangeul L (2006). Reduced diversity of faecal microbiota in Crohn's disease revealed by a metagenomic approach. Gut.

[64] Seksik P, Rigottier-Gois L, Gramet G, Sutren M, Pochart P, Marteau P (2003). Alterations of the dominant faecal bacterial groups in patients with Crohn's disease of the colon. Gut.

[65] Friedrich M, Pohin M, Powrie F (2019). Cytokine networks in the pathophysiology of inflammatory bowel disease. Immunity.

[66] Dickson RP, Erb-Downward JR, Martinez FJ, Huffnagle GB (2016). The microbiome and the respiratory tract. Annu. Rev. Physiol..

[67] Man WH, de Steenhuijsen Piters WA, Bogaert D (2017). The microbiota of the respiratory tract: gatekeeper to respiratory health. Nat. Rev. Microbiol..

[68] Mims JW (2015). Asthma: definitions and pathophysiology. Int. Forum Allergy Rhinol. 5 Suppl.

[69] Barcik W, Boutin RCT, Sokolowska M, Finlay BB (2020). The role of lung and gut microbiota in the pathology of asthma. Immunity.

[70] Thomas T, Gilbert J, Meyer F (2012). Metagenomics - a guide from sampling to data analysis. Microb. Inform. Exp..

[71] Paudel KR, Dharwal V, Patel VK, Galvao I, Wadhwa R, Malyla V (2020). Role of lung microbiome in innate immune response associated with chronic lung diseases. Front. Med (Lausanne).

[72] Azim A, Green B, Lau L, Rupani H, Jayasekera N, Bruce K (2021). Peripheral airways type 2 inflammation, neutrophilia and microbial dysbiosis in severe asthma. Allergy.

[73] Denner DR, Sangwan N, Becker JB, Hogarth DK, Oldham J, Castillo J (2016). Corticosteroid therapy and airflow obstruction influence the bronchial microbiome, which is distinct from that of bronchoalveolar lavage in asthmatic airways. J. Allergy Clin. Immunol..

[74] Boers E, Barrett M, Su JG, Benjafield AV, Sinha S, Kaye L (2023). Global burden of chronic obstructive pulmonary disease through 2050. JAMA Netw Open.

[75] Natalini JG, Singh S, Segal LN (2023). The dynamic lung microbiome in health and disease. Nat. Rev. Microbiol..

[76] Weeks JR, Staples KJ, Spalluto CM, Watson A, Wilkinson TMA (2021). The role of non-typeable *Haemophilus influenzae* biofilms in chronic obstructive pulmonary disease. Front. Cell. Infect. Microbiol..

[77] Klomp M, Ghosh S, Mohammed S, Nadeem Khan M (2021). From virus to inflammation, how influenza promotes lung damage. J. Leukoc. Biol..

[78] Lalbiaktluangi C, Yadav MK, Singh PK, Singh A, Iyer M, Vellingiri B (2023). A cooperativity between virus and bacteria during respiratory infections. Front. Microbiol..

[79] Sarda C, Palma P, Rello J (2019). Severe influenza: overview in critically ill patients. Curr. Opin. Crit. Care.

[80] Pacheco GA, Galvez NMS, Soto JA, Andrade CA, Kalergis AM. 2021. Bacterial and viral coinfections with the human respiratory syncytial virus. *Microorganisms*. **9**. 10.3390/microorganisms9061293 34199284 PMC8231868

[81] Edouard S, Million M, Bachar D, Dubourg G, Michelle C, Ninove L (2018). The nasopharyngeal microbiota in patients with viral respiratory tract infections is enriched in bacterial pathogens. Eur. J. Clin. Microbiol. Infect. Dis..

[82] Bouquet J, Tabor DE, Silver JS, Nair V, Tovchigrechko A, Griffin MP (2020). Microbial burden and viral exacerbations in a longitudinal multicenter COPD cohort. Respir. Res..

[83] Cyprian F, Sohail MU, Abdelhafez I, Salman S, Attique Z, Kamareddine L (2021). SARS-CoV-2 and immune-microbiome interactions: lessons from respiratory viral infections. Int. J. Infect. Dis..

[84] Smith N, Goncalves P, Charbit B, Grzelak L, Beretta M, Planchais C (2021). Distinct systemic and mucosal immune responses during acute SARS-CoV-2 infection. Nat. Immunol..

[85] Zhu B, Tao Z, Edupuganti L, Serrano MG, Buck GA (2022). Roles of the microbiota of the female reproductive tract in gynecological and reproductive health. Microbiol. Mol. Biol. Rev..

[86] Hillier SL, Lau RJ (1997). Vaginal microflora in postmenopausal women who have not received estrogen replacement therapy. Clin. Infect. Dis. 25 Suppl.

[87] Ravel J, Gajer P, Abdo Z, Schneider GM, Koenig SS, McCulle SL (2011). Vaginal microbiome of reproductive-age women. Proc. Natl. Acad. Sci. USA 108 Suppl.

[88] Yuk YS, Choi JE, Kim JK (2021). Age and sex trends of *Gardnerella vaginalis* infection in patients with sexually transmitted infections in Korea. Iran J. Microbiol..

[89] Dizzell S, Nazli A, Reid G, Kaushic C (2019). Protective effect of probiotic bacteria and estrogen in preventing HIV-1-mediated impairment of epithelial barrier integrity in female genital tract. Cells.

[90] Han Y, Liu Z, Chen T (2021). Role of vaginal microbiota dysbiosis in gynecological diseases and the potential interventions. Front. Microbiol..

[91] Hufstetler K, Llata E, Miele K, Quilter LAS (2024). Clinical updates in sexually transmitted infections, 2024. J. Womens Health (Larchmt).

[92] Yonke N, Aragon M, Phillips JK (2022). Chlamydial and gonococcal infections: screening, diagnosis, and treatment. Am. Fam. Phys..

[93] Brotman RM, Klebanoff MA, Nansel TR, Yu KF, Andrews WW, Zhang J (2010). Bacterial vaginosis assessed by gram stain and diminished colonization resistance to incident gonococcal, chlamydial, and trichomonal genital infection. J. Infect. Dis..

[94] Drell T, Lillsaar T, Tummeleht L, Simm J, Aaspollu A, Vain E (2013). Characterization of the vaginal micro- and mycobiome in asymptomatic reproductive-age Estonian women. PLoS One.

[95] Mirzaei R, Kavyani B, Nabizadeh E, Kadkhoda H, Asghari Ozma M, Abdi M (2023). Microbiota metabolites in the female reproductive system: focused on the short-chain fatty acids. Heliyon.

[96] Darbandi A, Asadi A, Mahdizade Ari M, Ohadi E, Talebi M, Halaj Zadeh M (2022). Bacteriocins: properties and potential use as antimicrobials. J. Clin. Lab Anal..

[97] Lenz JD, Dillard JP (2018). Pathogenesis of *Neisseria gonorrhoeae* and the host defense in ascending infections of human fallopian tube. Front. Immunol..

[98] Timmerman MM, Shao JQ, Apicella MA (2005). Ultrastructural analysis of the pathogenesis of *Neisseria gonorrhoeae* endometrial infection. Cell Microbiol..

[99] Masson L, Mlisana K, Little F, Werner L, Mkhize NN, Ronacher K (2014). Defining genital tract cytokine signatures of sexually transmitted infections and bacterial vaginosis in women at high risk of HIV infection: a cross-sectional study. Sex Transm. Infect..

[100] Kyrgiou M, Moscicki AB (2022). Vaginal microbiome and cervical cancer. Semin. Cancer Biol..

[101] Hong S, Choi DW, Kim HN, Park CG, Lee W, Park HH (2020). Protein-based nanoparticles as drug delivery systems. Pharmaceutics.

[102] Martinez-Lopez AL, Pangua C, Reboredo C, Campion R, Morales-Gracia J, Irache JM (2020). Protein-based nanoparticles for drug delivery purposes. Int. J. Pharm..

[103] Kiran F, Afzaal M, Shahid H, Saeed F, Ahmad A, Ateeq H (2023). Effect of protein-based nanoencapsulation on viability of probiotic bacteria under hostile conditions. Int. J. Food Prop..

[104] Devarajan A, Mudgil P, Aldhaheri F, Hamed F, Dhital S, Maqsood S (2022). Camel milk-derived probiotic strains encapsulated in camel casein and gelatin complex microcapsules: stability against thermal challenge and simulated gastrointestinal digestion conditions. J. Dairy Sci..

[105] Bernocchi B, Carpentier R, Lantier I, Ducournau C, Dimier-Poisson I, Betbeder D (2016). Mechanisms allowing protein delivery in nasal mucosa using NPL nanoparticles. J. Control. Release.

[106] Ou B, Yang Y, Lv H, Lin X, Zhang M (2023). Current progress and challenges in the study of adjuvants for oral vaccines. BioDrugs.

[107] Liu Z, Xiang C, Zhao X, Aizawa T, Niu R, Zhao J (2024). Regulation of dynamic spatiotemporal inflammation by nanomaterials in spinal cord injury. J. Nanobiotechnol..

[108] Bossi AM, Casella S, Stranieri C, Marinangeli A, Bucciarelli A, Fratta Pasini AM (2025). Protein-based molecular imprinting: gelatin nanotraps for interleukin-6 sequestration in inflammation cell models. Trends Biotechnol..

[109] Gao J, Li J, Luo Z, Wang H, Ma Z (2024). Nanoparticle-based drug delivery systems for inflammatory bowel disease treatment. Drug Des. Devel. Ther..

[110] Cojocaru E, Petris OR, Cojocaru C (2024). Nanoparticle-based drug delivery systems in inhaled therapy: improving respiratory medicine. Pharmaceuticals (Basel).

[111] Zhang T, Li L, Chunta S, Wu W, Chen Z, Lu Y (2023). Enhanced oral bioavailability from food protein nanoparticles: a mini review. J. Control. Release.

[112] Yang G, Phua SZF, Bindra AK, Zhao Y (2019). Degradability and clearance of inorganic nanoparticles for biomedical applications. Adv. Mater..

[113] Ashfaq R, Rasul A, Asghar S, Kovacs A, Berko S, Budai-Szucs M (2023). Lipid nanoparticles: an effective tool to improve the bioavailability of nutraceuticals. Int. J. Mol. Sci..

[114] Mall J, Naseem N, Haider MF, Rahman MA, Khan S, Siddiqui SN. 2024. Nanostructured lipid carriers as a drug delivery system: a comprehensive review with therapeutic applications. *Intelligent Pharm.*, In Press. 10.1016/j.ipha.2024.09.005

[115] Xu L, Wang X, Liu Y, Yang G, Falconer RJ, Zhao C-X (2022). Lipid nanoparticles for drug delivery. Adv. NanoBiomed Res..

[116] Han M, Shen N, Tan W, Wang X, Liu Y, Liang J (2024). Layer-by-layer coated probiotics with chitosan and liposomes exhibit enhanced therapeutic effects for DSS-induced colitis in mice. Int. J. Biol. Macromol..

[117] Hou X, Zaks T, Langer R, Dong Y (2021). Lipid nanoparticles for mRNA delivery. Nat. Rev. Mater..

[118] Anderluzzi G, Lou G, Woods S, Schmidt ST, Gallorini S, Brazzoli M (2022). The role of nanoparticle format and route of administration on self-amplifying mRNA vaccine potency. J. Control. Release.

[119] Mohammadi G, Sotoudehnia Koranni Z, Jebali A (2021). The oral vaccine based on self-replicating RNA lipid nanoparticles can simultaneously neutralize both SARS-CoV-2 variants alpha and delta. Int Immunopharmacol..

[120] Ball RL, Bajaj P, Whitehead KA (2018). Oral delivery of siRNA lipid nanoparticles: fate in the GI tract. Sci. Rep..

[121] Garbati P, Picco C, Magrassi R, Signorello P, Cacopardo L, Dalla Serra M (2024). Targeting the gut: a systematic review of specific drug nanocarriers. Pharmaceutics.

[122] Khan W, Abtew E, Modani S, Domb AJ (2018). Polysaccharide based nanoparticles. Israel J. Chem..

[123] Wang N, Zhang C, Li H, Zhang D, Wu J, Li Y (2024). Addition of Canna edulis starch and starch nanoparticles to stabilized Pickering emulsions: *in vitro* digestion and fecal fermentation. Int. J. Biol. Macromol..

[124] Qiu L, Shen R, Wei L, Xu S, Xia W, Hou Y (2023). Designing a microbial fermentation-functionalized alginate microsphere for targeted release of 5-ASA using nano dietary fiber carrier for inflammatory bowel disease treatment. J. Nanobiotechnol..

[125] Wang M, Cha R, Hao W, Du R, Zhang P, Hu Y (2022). Nanocrystalline cellulose cures constipation via gut microbiota metabolism. ACS Nano.

[126] Pifferi C, Fuentes R, Fernandez-Tejada A (2021). Natural and synthetic carbohydrate-based vaccine adjuvants and their mechanisms of action. Nat. Rev. Chem..

[127] Hammerman MR, Rogers S, Morrissey JJ, Gavin JR (1986). Phorbol ester-stimulated phosphorylation of basolateral membranes from canine kidney. Am. J. Physiol..

[128] Dreno B, Alexis A, Chuberre B, Marinovich M (2019). Safety of titanium dioxide nanoparticles in cosmetics. J. Eur. Acad. Dermatol. Venereol. 33 Suppl.

[129] Baranowska-Wojcik E (2021). Factors conditioning the potential effects TiO2 NPs exposure on human microbiota: a mini-review. Biol. Trace Elem. Res..

[130] Jiang Y, Qi S, Mao C (2025). Polysaccharide nanoparticles as potential immune adjuvants: mechanism and function. Acta Pharm. Sinica B..

[131] Ahmed FY, Aly UF, Abd El-Baky RM, Waly N (2021). Effect of titanium dioxide nanoparticles on the expression of efflux pump and quorum-sensing genes in MDR *Pseudomonas aeruginosa* isolates. Antibiotics (Basel).

[132] de Dicastillo CL, Patino C, Galotto MJ, Vasquez-Martinez Y, Torrent C, Alburquenque D (2019). Novel hollow titanium dioxide nanospheres with antimicrobial activity against resistant bacteria. Beilstein J. Nanotechnol..

[133] Limage R, Tako E, Kolba N, Guo Z, Garcia-Rodriguez A, Marques CNH (2020). TiO(2) nanoparticles and commensal bacteria alter mucus layer thickness and composition in a gastrointestinal tract model. Small.

[134] Grondin JA, Kwon YH, Far PM, Haq S, Khan WI (2020). Mucins in intestinal mucosal defense and inflammation: learning from clinical and experimental studies. Front. Immunol..

[135] Racovita AD (2022). Titanium dioxide: structure, impact, and toxicity. Int. J. Environ. Res. Public Health.

[136] Ripolles-Avila C, Martinez-Garcia M, Hascoët A-S, Rodríguez-Jerez JJ (2019). Bactericidal efficacy of UV activated TiO2 nanoparticles against Gram-positive and Gram-negative bacteria on suspension. CyTA-J. Food.

[137] Veranth JM, Kaser EG, Veranth MM, Koch M, Yost GS (2007). Cytokine responses of human lung cells (BEAS-2B) treated with micron-sized and nanoparticles of metal oxides compared to soil dusts. Part Fibre Toxicol..

[138] Kroll A, Pillukat MH, Hahn D, Schnekenburger J (2012). Interference of engineered nanoparticles with in vitro toxicity assays. Arch. Toxicol..

[139] Tardelli JDC, Bagnato VS, Reis ACD (2023). Bacterial adhesion strength on titanium surfaces quantified by atomic force microscopy: a systematic review. Antibiotics (Basel).

[140] Warheit DB, Brown SC, Donner EM (2015). Acute and subchronic oral toxicity studies in rats with nanoscale and pigment grade titanium dioxide particles. Food Chem. Toxicol..

[141] Dove AS, Dzurny DI, Dees WR, Qin N, Nunez Rodriguez CC, Alt LA (2022). Silver nanoparticles enhance the efficacy of aminoglycosides against antibiotic-resistant bacteria. Front. Microbiol..

[142] Sondi I, Salopek-Sondi B (2004). Silver nanoparticles as antimicrobial agent: a case study on *E. coli* as a model for Gram-negative bacteria. J. Colloid Interface Sci..

[143] Jung WK, Koo HC, Kim KW, Shin S, Kim SH, Park YH (2008). Antibacterial activity and mechanism of action of the silver ion in *Staphylococcus aureus* and *Escherichia coli*. Appl. Environ. Microbiol..

[144] Arakawa H, Neault JF, Tajmir-Riahi HA (2001). Silver(I) complexes with DNA and RNA studied by Fourier transform infrared spectroscopy and capillary electrophoresis. Biophys. J..

[145] Rodrigues AS, Batista JGS, Rodrigues MAV, Thipe VC, Minarini LAR, Lopes PS (2024). Advances in silver nanoparticles: a comprehensive review on their potential as antimicrobial agents and their mechanisms of action elucidated by proteomics. Front. Microbiol..

[146] Mohanta YK, Biswas K, Jena SK, Hashem A, Abd Allah EF, Mohanta TK (2020). Corrigendum: anti-biofilm and antibacterial activities of silver nanoparticles synthesized by the reducing activity of pytoconstituents present in the Indian medicinal plants. Front. Microbiol..

[147] Chen H, Zhao R, Wang B, Cai C, Zheng L, Wang H (2017). The effects of orally administered Ag, TiO2 and SiO2 nanoparticles on gut microbiota composition and colitis induction in mice. NanoImpact..

[148] Mijnendonckx K, Leys N, Mahillon J, Silver S, Van Houdt R (2013). Antimicrobial silver: uses, toxicity and potential for resistance. Biometals.

[149] O'Shaughnessy M, Sheils O, Baird AM (2023). The lung microbiome in COPD and lung cancer: exploring the potential of metalbased drugs. Int. J. Mol. Sci..

[150] Carvalho-Silva JM, Reis ACD (2024). Anti-inflammatory action of silver nanoparticles *in vivo*: systematic review and meta-analysis. Heliyon.

[151] Carlson C, Hussain SM, Schrand AM, Braydich-Stolle LK, Hess KL, Jones RL (2008). Unique cellular interaction of silver nanoparticles: size-dependent generation of reactive oxygen species. J. Phys. Chem. B..

[152] Martin-Faivre L, Prince L, Cornu C, Villeret B, Sanchez-Guzman D, Rouzet F (2025). Pulmonary delivery of silver nanoparticles prevents influenza infection by recruiting and activating lymphoid cells. Biomaterials.

[153] Gliga AR, Di Bucchianico S, Lindvall J, Fadeel B, Karlsson HL (2018). RNA-sequencing reveals long-term effects of silver nanoparticles on human lung cells. Sci. Rep..

[154] Chen X, Argandona SM, Melle F, Rampal N, Fairen-Jimenez D (2024). Advances in surface functionalization of next-generation metal-organic frameworks for biomedical applications: design, strategies, and prospects. Chem.

[155] Roberfroid MB (2000). Prebiotics and probiotics: are they functional foods?. Am. J. Clin. Nutr..

[156] Zhou P, Chen C, Patil S, Dong S (2024). Unveiling the therapeutic symphony of probiotics, prebiotics, and postbiotics in gut-immune harmony. Front. Nutr..

[157] Han S, Lu Y, Xie J, Fei Y, Zheng G, Wang Z (2021). Probiotic gastrointestinal transit and colonization after oral administration: a long journey. Front. Cell. Infect. Microbiol..

[158] Sun Q, Yin S, He Y, Cao Y, Jiang C (2023). Biomaterials and encapsulation techniques for probiotics: current status and future prospects in biomedical applications. Nanomaterials (Basel).

[159] Arratia-Quijada J, Nuño K, Ruíz-Santoyo V, Andrade-Espinoza BA (2024). Nano-encapsulation of probiotics: need and critical considerations to design new non-dairy probiotic products. J. Funct. Foods.

[160] Edo GI, Mafe AN, Razooqi NF, Umelo EC, Gaaz TS, Isoje EF (2025). Advances in bio-polymer coatings for probiotic microencapsulation: chitosan and beyond for enhanced stability and controlled release. Des. Monomers Polym..

[161] Wang X, Gao S, Yun S, Zhang M, Peng L, Li Y (2022). Microencapsulating alginate-based polymers for probiotics delivery systems and their application. Pharmaceuticals (Basel).

[162] Dafe A, Etemadi H, Dilmaghani A, Mahdavinia GR (2017). Investigation of pectin/starch hydrogel as a carrier for oral delivery of probiotic bacteria. Int. J. Biol. Macromol..

[163] Zhou L, Huang Y, Wang D, Yuan T, Song G, Gong J (2024). Microencapsulation of *Lactobacillus sakei* and *Lactobacillus rhamnosus* in whey protein isolate and sodium hyaluronate for potential food-grade probiotic delivery system. Food Biosci..

[164] Elzoghby AO, El-Fotoh WS, Elgindy NA (2011). Casein-based formulations as promising controlled release drug delivery systems. J. Control. Release.

[165] Wang L, Zhong X, Li S, Liu X, Wang K, Cai R (2024). Probiotics encapsulated by gelatin and hyaluronic acid via layer-by-layer assembly technology for enhanced viability. Food Hydrocoll..

[166] Azeem M, Saeed F, Afzaal M, Ateeq H, Ahmad A, Liaqat A (2023). Encapsulation of probiotics in solid lipid micro particle for improved viability and stability under stressed conditions. Int. J. Food Prop..

[167] Han M, Yang S, Song J, Gao Z (2024). Layer-by-layer coated probiotics with chitosan and liposomes demonstrate improved stability and antioxidant properties in vitro. Int. J. Biol. Macromol..

[168] Patarroyo JL, Florez-Rojas JS, Pradilla D, Valderrama-Rincon JD, Cruz JC, Reyes LH (2020). Formulation and characterization of gelatin-based hydrogels for the encapsulation of *Kluyveromyces lactis*-applications in packed-bed reactors and probiotics delivery in humans. Polymers (Basel).

[169] Contreras BG, De Vuyst L, Devreese B, Busanyova K, Raymaeckers J, Bosman F (1997). Isolation, purification, and amino acid sequence of lactobin A, one of the two bacteriocins produced by Lactobacillus amylovorus LMG P-13139. Appl. Environ. Microbiol..

[170] Tichaczek PS, Nissen-Meyer J, Nes IF, Vogel RF, Hammes WP (1992). Characterization of the bacteriocins curvacin A from *Lactobacillus curvatus* LTH1174 and sakacin P from *L. sake* LTH673. Syst. Appl. Microbiol..

[171] Tichaczek PS, Vogel RF, Hammes WP (1993). Cloning and sequencing of curA encoding curvacin A, the bacteriocin produced by Lactobacillus curvatus LTH1174. Arch. Microbiol..

[172] BENMOUNA Z, VALDIVIA E, MONTALBÁN-LÓPEZ M, DALACHE F, ZADI-KARAM H, KARAM N-E (2024). Probiotic potential of *Enterococcus* strains with multiple enterocin-encoding genes. Not. Sci. Biol..

[173] Todorov SD, Dioso CM, Liong MT, Nero LA, Khosravi-Darani K, Ivanova IV (2022). Beneficial features of pediococcus: from starter cultures and inhibitory activities to probiotic benefits. World J. Microbiol. Biotechnol..

[174] Mandal SM, Silva ON, Franco OL (2014). Recombinant probiotics with antimicrobial peptides: a dual strategy to improve immune response in immunocompromised patients. Drug Discov. Today.

[175] You J, Dong H, Mann ER, Knight SC, Yaqoob P (2014). Probiotic modulation of dendritic cell function is influenced by ageing. Immunobiology.

[176] Yoha KS, Nida S, Dutta S, Moses JA, Anandharamakrishnan C (2022). Targeted delivery of probiotics: perspectives on research and commercialization. Probiotics Antimicrob. Proteins.

[177] Kaewarsar E, Chaiyasut C, Lailerd N, Makhamrueang N, Peerajan S, Sirilun S (2023). Optimization of mixed inulin, fructooligosaccharides, and galactooligosaccharides as prebiotics for stimulation of probiotics growth and function. Foods.

[178] Pandey KR, Naik SR, Vakil BV (2015). Probiotics, prebiotics and synbiotics- a review. J. Food Sci. Technol..

[179] Slavin J (2013). Fiber and prebiotics: mechanisms and health benefits. Nutrients.

[180] Zhu R, Yuan W, Xia A, Sun X, Yan W, Wu T (2024). Inulin‐based nanoparticle modulates gut microbiota and mmune microenvironment for improving colorectal cancer therapy. Adv. Funct. Mater..

[181] Wang G, Sun W, Pei X, Jin Y, Wang H, Tao W (2021). Galactooligosaccharide pretreatment alleviates damage of the intestinal barrier and inflammatory responses in LPS-challenged mice. Food Funct..

[182] Rashidinejad A, Bahrami A, Rehman A, Rezaei A, Babazadeh A, Singh H (2022). Co-encapsulation of probiotics with prebiotics and their application in functional/synbiotic dairy products. Crit. Rev. Food Sci. Nutr..

[183] Durazzo A, Nazhand A, Lucarini M, Atanasov AG, Souto EB, Novellino E (2020). An updated overview on nanonutraceuticals: focus on nanoprebiotics and nanoprobiotics. Int. J. Mol. Sci..

[184] Senthil Kumar S, Sheik Mohideen S (2024). Chitosan-coated probiotic nanoparticles mitigate acrylamide-induced toxicity in the Drosophila model. Sci. Rep..

[185] Luan Q, Zhou W, Zhang H, Bao Y, Zheng M, Shi J (2018). Cellulose-based composite macrogels from cellulose fiber and cellulose nanofiber as intestine delivery vehicles for probiotics. J. Agric. Food Chem..

[186] Maleki O, Khaledabad MA, Amiri S, Asl AK, Makouie S (2020). Microencapsulation of *Lactobacillus rhamnosus* ATCC 7469 in whey protein isolate-crystalline nanocellulose-inulin composite enhanced gastrointestinal survivability. LWT.

[187] Saadatzadeh A, Atyabi F, Fazeli MR, Dinarvand R, Jamalifar H, Abdolghaffari AH (2012). Biochemical and pathological evidences on the benefit of a new biodegradable nanoparticles of probiotic extract in murine colitis. Fundam Clin. Pharmacol..

[188] Alkushi AG, Elazab ST, Abdelfattah-Hassan A, Mahfouz H, Salem GA, Sheraiba NI (2022). Multi-strain-probiotic-loaded nanoparticles reduced colon inflammation and orchestrated the expressions of tight junction, NLRP3 inflammasome and caspase-1 genes in DSS-induced colitis model. Pharmaceutics.

[189] Lopes SA, Roque-Borda CA, Duarte JL, Di Filippo LD, Borges Cardoso VM, Pavan FR (2023). Delivery strategies of probiotics from nano- and microparticles: trends in the treatment of inflammatory bowel disease-An overview. Pharmaceutics.

[190] Kumar N, Tyagi N, Mehan S, Singh AP (2024). Formulation of solid lipid nanoparticles loaded with rosiglitazone and probiotic: optimization and in-vitro characterization. Recent Pat. Nanotechnol..

[191] Rezaee P, Kermanshahi R, Katouli M (2014). Prebiotics decrease the antibacterial effect of nano silver and nano TiO 2 particles against probiotic bacteria of food. Curr. Nutr. Food Sci..

[192] Catto C, Garuglieri E, Borruso L, Erba D, Casiraghi MC, Cappitelli F (2019). Impacts of dietary silver nanoparticles and probiotic administration on the microbiota of an in-vitro gut model. Environ. Pollut..

[193] Zhao Y, Tang Y, Chen L, Lv S, Liu S, Nie P (2020). Restraining the TiO(2) nanoparticles-induced intestinal inflammation mediated by gut microbiota in juvenile rats via ingestion of Lactobacillus rhamnosus GG. Ecotoxicol. Environ. Saf..

[194] Fu J, Liu X, Cui Z, Zheng Y, Jiang H, Zhang Y (2023). Probiotic-based nanoparticles for targeted microbiota modulation and immune restoration in bacterial pneumonia. Natl. Sci. Rev..

[195] Mei Z, Li D (2022). The role of probiotics in vaginal health. Front. Cell. Infect. Microbiol..

[196] Wei G, Liu Q, Wang X, Zhou Z, Zhao X, Zhou W (2023). A probiotic nanozyme hydrogel regulates vaginal microenvironment for Candida vaginitis therapy. Sci. Adv..

[197] Silva JA, De Gregorio PR, Rivero G, Abraham GA, Nader-Macias MEF (2021). Immobilization of vaginal *Lactobacillus* in polymeric nanofibers for its incorporation in vaginal probiotic products. Eur. J. Pharm. Sci..

[198] Chandrashekhar P, Minooei F, Arreguin W, Masigol M, Steinbach-Rankins JM (2021). Perspectives on existing and novel alternative intravaginal probiotic delivery methods in the context of bacterial vaginosis infection. AAPS J..

[199] Mastromarino P, Vitali B, Mosca L (2013). Bacterial vaginosis: a review on clinical trials with probiotics. New Microbiol..

[200] Zuniga Vinueza AM (2024). Probiotics for the prevention of vaginal infections: a systematic review. Cureus.

